# The Effect of Silica Nanoparticles on Human Corneal Epithelial Cells

**DOI:** 10.1038/srep37762

**Published:** 2016-11-23

**Authors:** Joo-Hee Park, Hyejoong Jeong, Jinkee Hong, Minwook Chang, Martha Kim, Roy S. Chuck, Jimmy K. Lee, Choul-Yong Park

**Affiliations:** 1 Department of Ophthalmology, Dongguk University, Ilsan Hospital, Goyang, South Korea; 2 School of Chemical Engineering and Material Science, Chung-Ang University, Seoul, South Korea; 3 Department of Ophthalmology and Visual Sciences, Montefiore Medical Center, Albert Einstein College of Medicine, Bronx, NY, USA

## Abstract

Ocular drug delivery is an interesting field in current research. Silica nanoparticles (SiNPs) are promising drug carriers for ophthalmic drug delivery. However, little is known about the toxicity of SiNPs on ocular surface cells such as human corneal epithelial cells (HCECs). In this study, we evaluated the cytotoxicity induced by 50, 100 and 150 nm sizes of SiNPs on cultured HCECs for up to 48 hours. SiNPs were up-taken by HCECs inside cytoplasmic vacuoles. Cellular reactive oxygen species generation was mildly elevated, dose dependently, with SiNPs, but no significant decrease of cellular viability was observed up to concentrations of 100 μg/ml for three different sized SiNPs. Western blot assays revealed that both cellular autophagy and mammalian target of rapamycin (mTOR) pathways were activated with the addition of SiNPs. Our findings suggested that 50, 100 and 150 nm sized SiNPs did not induce significant cytotoxicity in cultured HCECs.

The cornea is typically the major route of intraocular transport of topically applied drugs^1^. Corneal epithelial cells constitute the outermost mechanical barrier of the ocular surface^2^. These cells are replenished periodically in every 2 weeks by newly differentiated epithelial cells from the limbal area^2^,^3^. As a most surface layer, corneal epithelial cells are continuously exposed to the outer atmosphere, therefore, they provide the first line of defense against foreign materials invading the ocular surface^2^. This protective role of corneal epithelial cells, on the other hand, sometimes serves as a mechanical barrier for ocular penetration of topically administered medication^1^.

To enhance ocular drug penetration, nanoparticle based drug delivery systems have been intensively investigated with promising results^4^,^5^,^6^. Amorphous silica nanoparticles (SiNPs) are some of the most promising nanoparticle systems for ocular drug delivery. SiNPs have stable chemical structures, large surface to volume ratios, ease of surface modification and tolerable biodegradability^7^. Due to these physical properties, biomedical applications of SiNPs have been intensively investigated^7^,^8^. Recent study suggests that small sized (50 nm) silica nanoparticles are readily permeable into de-epithelialized cornea^9^.

However, cytotoxicity is the most significant issue with SiNPs. It is known that the cellular toxicity and biological effect of SiNPs are largely dependent on the size and concentration of the SiNPs^10^,^11^. In addition, different cell types have shown different susceptibility and patterns of SiNPs nanotoxicity^11^,^12^. Recently, several studies have demonstrated that SiNPs have no direct cytotoxicity on retinal endothelial cells and retinal neuronal tissue^13^. However, the nanotoxicity of SiNPs on corneal epithelial cells is not fully studied yet although these cells are the first encounters when SiNPs are topically administered for ocular therapy.

Herein, monodisperse and non-porous SiNPs with diameters of 50, 100 and 150 nm were employed to investigate how particle size and concentration affect the biological activities of human corneal epithelial cells. The effect of the size and concentration of SiNPs on biological responses including cellular viability, reactive oxygen species (ROS) generation and autophagy were evaluated. In addition, the effect of SiNPs on the upstream cellular proliferative pathway, the mammalian target of rapamycin (mTOR) pathway, was investigated.

## Results

### Characterization of SiNPs

**Figure 1:**
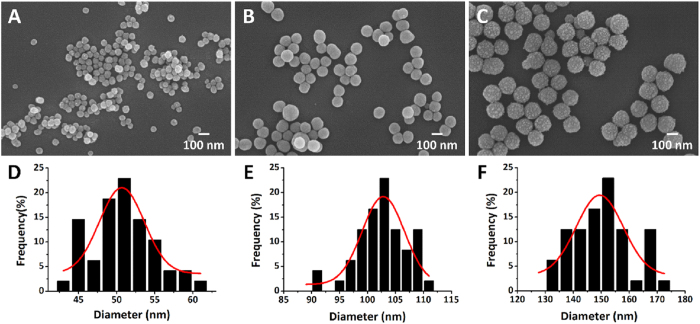
Morphologies of SiNPs were observed by SEM. (**A**) 50, (**B**) 100, (**C**) 150 nm. Size distribution graphs corresponding to the above SEM images are shown: (**D**) 50, (**E**) 100, (**F**) 150 nm.

We characterized the prepared SiNPs with three different sizes. The morphology of each SiNPs was observed using SEM and size distribution graphs were obtained from the micrographs (Fig. 1). From the results, we confirmed spherical shapes with uniform sizes for all the SiNPs. The numerical data for mean size and dispersity are summarized in Table 1. The mean sizes of the SiNPs weree 50.68, 102.81, and 149.41 nm according to the size distribution graphs in Fig. 1. The dispersity of nanoparticles is determined on the basis of the coefficient of variation: Nanoparticles with under 5% coefficient of variation are defined as monodispersed nanoparticles and those with under 15% are defined as near-monodispersed nanoparticles^14^. From Table 1, we determined that the prepared SiNPs were almost monodispersed since the dispersity was in the range of 3 to 5%. To identify the stability of the SiNPs in different aqueous solutions, we investigated the zeta potential. This is an electric charge at the shear plane, which is a thin layer between the particle surface and liquid. Zeta potential is useful to indicate the stability of the colloidal suspension. It is accepted that when the zeta potential is |**30**| mV and optimally higher than |**60**| mV, particles are fully stabilized by electrostatic charges and are less likely to be flocculated with each other due to electrostatic repulsion^11^,^15^. In distilled water, SiNPs have good stability and dispersion with higher zeta potentials over −50 mV. On the contrary, SiNPs dispersed in DPBS showed lower zeta potential close to a neutral charge. This is because the negative charge of SiNPs is offset by various salts in DPBS. From this result, we could predict that the charges of the SiNPs are almost neutral in cell culture media and are prone to agglomerate together in cell culture.

### Intracellular distribution of SiNPs

**Figure 2:**
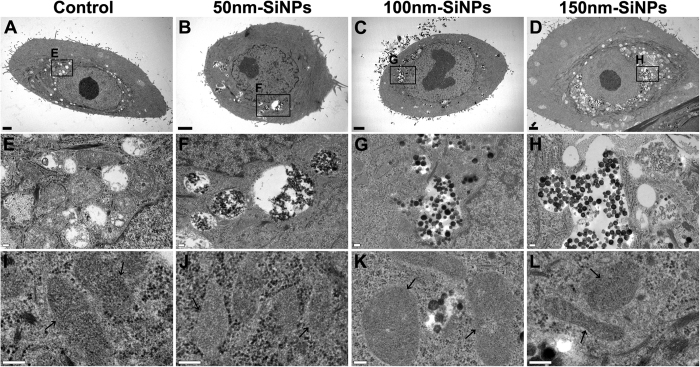
Cellular uptake of SiNPs in HCECs evaluated by transmission electron microscope. HCECs were cultured with various sizes of SiNPs for 24 h (**A**,**B**,**C** and **D**). SiNPs were mainly accumulated in cytoplasmic vesicles (**F**,**G** and **H**) while no SiNPs were observed in control (**E**). No nuclear entry of SiNPs was observed. Mitochondria remained intact with no visible damage on the structure (**I** to **L**). (**A**,**E** and **I)** negative control with no SiNPs; (**B**,**F** and **J**) 50 nm SiNP added (100 μg/mL); (**C**,**G** and **K**) 100 nm SiNP added (100 μg/mL); (**D**,**H** and **L**) 150 nm SiNP added (100 μg/mL).

Internalization and the intracellular distribution of SiNPs were evaluated by TEM. SiNPs were localized mainly in cytoplasmic vesicles around the nucleus (Fig. 2). Small amounts of SiNPs were found to exist outside the vesicles and were observed in the cytoplasmic matrix. However, no SiNPs were observed inside either the nucleus or mitochondria. Neither mitochondrial damage nor nuclear membrane damage was observed (Fig. 2).

### Oxidative stress induced by SiNPs on HCECs

**Figure 3:**
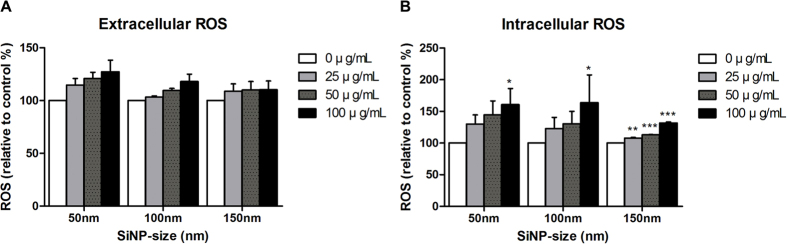
Induced reactive oxygen species (ROS) in human corneal epithelial cells (HCECs) following treatment with three different sizes of silica nanoparticles (SiNPs). Extracellular (**A**) and intracellular (**B**) ROS levels. Values are measured as means ± SEM (n = 3) and are calculated as % from negative control (0 μgmL treated groups). P values were calculated compared to negative control. *p < 0.05, **p < 0.01, ***p < 0.001.

**Figure 4:**
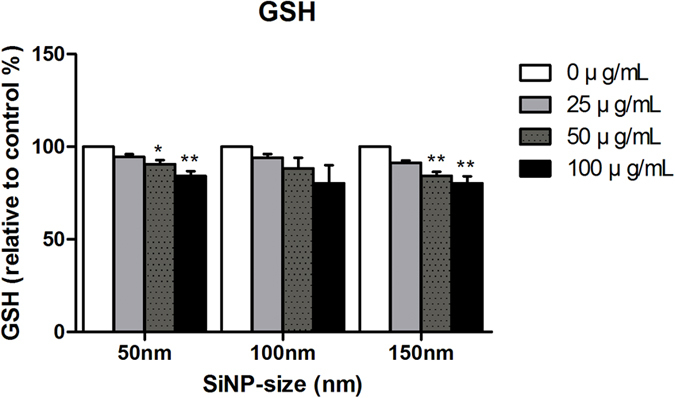
Total glutathione (GSH) level in HCECs after treatment with three sizes of SiNPs. Results are statistically calculated as mean ± SEM (n = 3) and are calculated as % from negative control (0 μg/mL treated groups). P values were calculated compared to negative control. *p < 0.05, **p < 0.01.

SiNPs increased both the intracellular and extracellular ROS levels of HCECs in a dose dependent manner (Fig. 3). This dose dependent ROS increase was observed in all three SiNPs sizes. However, intracellular ROS increase by 150 nm SiNP was significantly lower than that by 50 and 100 nm SiNPs (*p* values < 0.05). Similar findings were observed for extracellular ROS levels, but the difference was not significant. The expected inverse relationship between ROS and GSH was observed. With the increase of ROS, GSH level decreased accordingly (Fig. 4).

### Cellular Autophagy

**Figure 5:**
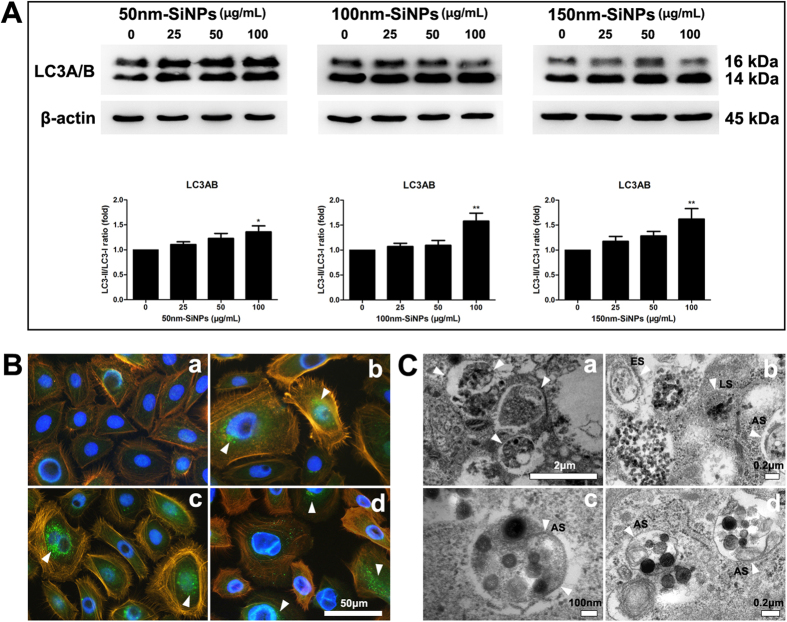
The effect of SiNPs on HCECs’ autophagy. (**A**) LC3A/B conversion in HCEC treated with SiNPs for 24 h. The expression levels for the autophagy signal, LC3A/B proteins, were measured by Western blot analysis. The inactive I form is 16 kDa and the active II form is 14 kDa. Densitometric analyses of western blots showed the increased expression of II form with higher concentration of SiNPs added. Values (mean ± SEM) are expressed as a percentage of the control and were obtained from three independent experiments; each independent experiment was performed in triplicate (*p < 0.05, **p < 0.01). (**B**) Immunocytochemical staining with LC3B antibody revealed the increased autophagy in HCECs with 100 μg/mL of 50 nm SiNP (b), 100 nm SiNP (c) and 150 nm SiNP (d) addition. White arrowheads indicated the cells with increased LC3B staining (green). DAPI stained nucleus with blue and orange represented F-actin. Negative control is HCECs with no SiNPs addition (a). (**C**) Transmission electron microscope (TEM) revealed some SiNPs inside amphisomes (AS), endosome (ES), and lysosome (LS) (white arrowheads in b, c and d). a: positive control of autophagosomes (white arrowheads) induced by incubation with 50 μM-chloroquine diphosphate for 24 h in HCECs.

We investigated the effect of SiNPs on the cellular autophagy system using the signal alteration of LC3A/B, the autophagy marker (Fig. 5A). With the activation of autophagy, LC3A/B II form increases relative to LC3A/B I form. All three sizes of SiNPs triggered significant expression of LC3A/B II proteins. The increased ratio of activated LC3A/B were more prominent with a high concentration (100 μg/mL) of SiNPs stimuli and reached up to 1.35 fold (50 nm SiNPs), 1.62 fold (100 nm SiNPs) and 1.63 fold (150 nm SiNPs). Increased LC3B proteins in cytoplasm with SiNPs addition were also demonstrated by immunocytochemistry. (Fig. 5B) Some of SiNPs were captured inside endosomes or amphisomes (Fig. 5C) and some were found in autophagosomes and lysosomes, which were characterized by double membranous vacuoles.

### Cellular viability, LDH and TUNEL assay

**Figure 6:**
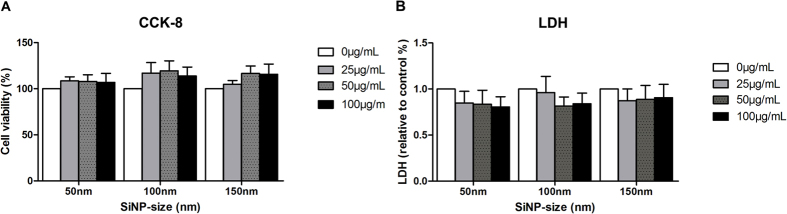
Cellular viability assay. Cellular viability using CCK-8 (**A**) and lactose dehydrogenase (LDH) after 24 h exposure to 25 μg/mL, 50 μg/mL, or 100 μg/mL of SiNP treated HCEC. No significant changes were observed with SiNPs. Quadruplicates of each treatment group were used in each independent experiment. Values are the mean ± SEM from four independent experiments.

**Figure 7:**
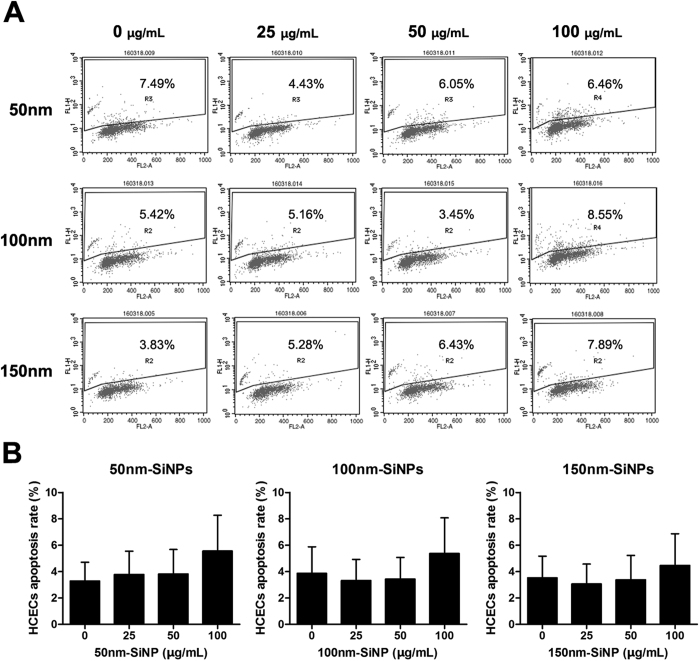
Apoptosis assay. Cellular apoptosis using the TUNEL assay following treatment with SiNPs for 24 h in HCECs. These representative dot plot figures show the most severe values (% of gated) for TUNEL positive labeled results obtained (n = 3) (**A**). The mean ± SEM for three independent experiments were calculated and are shown on the graph (**B**).

HCEC viability was not affected with the treatment of SiNPs (Fig. 6A) Accordingly, LDH level was unchanged with the treatment of SiNPs (Fig. 6B). The size and concentration of SiNPs did not affect the viability or the LDH levels significantly. In addition, TUNEL assay following 24 h treatment of SiNPs showed that none of the SiNPs sizes induced apoptosis (Fig. 7). Only 6.46, 8.55, and 7.89% of apoptotic cells were detected with the treatment of 100 μg/mL of 50, 100, 150 nm SiNPs, respectively. When considering 3.83~7.49% of apoptotic cells in the negative controls, the proportion of apoptotic cells with SiNPs treatment were considered to be in the normal range.

### mTOR pathway activation

**Figure 8:**
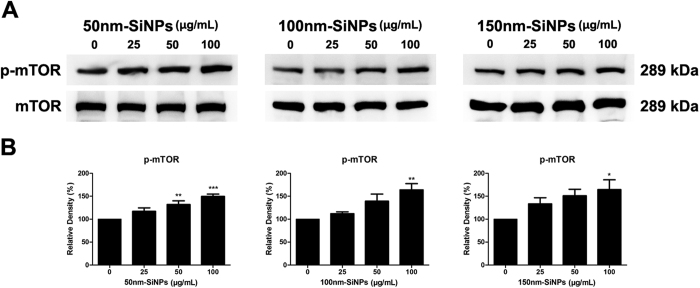
Effect of SiNPs on mTOR signaling. The expression levels of phosphorylated mTOR (p-mTOR) and mTOR detected by Western blot analysis (**A**), and relative densitometry (**B**) were calculated as a percentage of the control and all values (mean ± SEM) were obtained from three independent experiments; each independent experiment was performed in triplicate. *p < 0.05, **p < 0.01, ***p < 0.001.

SiNPs increased the mTOR pathway of HCEC. We measured the expression level of phosphorylated mTOR (p-mTOR) and mTOR (Fig. 8). The expression of p-mTOR increased 1.50, 1.64 and 1.64 fold with 100 μg/mL concentrations of 50 nm, 100 nm and 150 nm-SiNPs, respectively, when compared to the normal control. This suggests that the SiNPs used in this study triggered the cell survival pathway, mTOR signal transduction, which is consistent with the cellular viability assay.

## Discussion

In this study, we investigated the effect of three different sizes (50, 100, 150 nm) of SiNPs on cultured HCEC. As revealed by TEM study, SiNPs are localized mainly in the cytoplasm of HCEC. Although, all three SiNPs (up to 100 μg/ml concentration) induced a slight increase of intracellular ROS, the cellular viability and intracellular survival machineries such as mTOR pathway and autophagy remained intact.

It is known that ocular drug penetration is difficult and can be hindered by extrinsic and intrinsic ocular barriers such as tear film, mucus barrier, tight junction of the corneal epithelium, hydrophilicity of the corneal epithelium, and hydrophobicity of the corneal stroma^1^. Therefore, efficient ocular drugs should have both hydrophilic and hydrophobic properties and sizes small enough to pass through the above-mentioned barriers for successful intraocular penetration. Much of topically applied drug solution is lost via nasolacrimal and conjunctival vascular drainage before penetrating the cornea and only 1% or less of an eyedrop actually reaches the intraocular tissues^16^. To overcome poor drug penetration with topical administration in many cases, intravitreal injection is now becoming increasingly popular in clinical practice. However, intraocular injection increases the risk of intraocular infection, which may be sight threatening, and is also accompanied by pain and higher costs^17^. Therefore, topical medication may be more desirable, especially considering ease of instillation and safety. If a drug can penetrate the full thickness of cornea and reach the anterior chamber in high concentration, then further diffusion into the posterior chamber should occur easily. Recently, it was suggested that this long challenging problem could be overcome with the use of nano-based drug carriers^5^,^6^. Several recent reports of successful intraocular drugs or gene deliveries by various nanoparticles further increased such expectations^18^,^19^,^20^,^21^,^22^,^23^,^24^. The major advantage of nanoparticles is enhanced cellular uptake due to their small size.

SiNPs are some of the intensively investigated nanomaterials as promising drug carriers in various biomedical fields. The negative charge of SiNPs based on the presence of hydroxyl groups makes them more feasible for surface modification, which can control physicochemical, toxicological and pharmacological properties^7^,^8^. Although SiNPs-based drug delivery is not yet popular in the ocular system, there are previous reports that SiNPs themselves inhibited retinal and corneal angiogenesis *in vivo* and *in vitro*^13^,^25^. The antiangiogenic effect of SiNPs is very inspiring with regard to the development of new ocular drug delivery systems because many intractable ocular diseases are accompanied by neovascularization^26^. However, there remain some obstacles to overcome for successful use of SiNPs for ocular drug delivery. For example, Mun *et al*.^9^ reported that the corneal epithelium functions as a strong mechanical and chemical barrier to SiNPs penetration.

Despite many promising aspects of nanoparticles in clinical applications, significant concerns about nanotoxicity are still major limitations. The relatively poor knowledge about the exact mechanism of nanotoxicity may further enhance the safety concerns. Concerns related to SiNPs nanotoxicity have been raised before^27^. Systemic administration of high doses of SiNPs in animal models resulted in multiple organ damage^28^,^29^. In addition, SiNPs addition to culture media significantly increased cellular ROS production and intracellular Ca^2+^ accumulation^30^. Besides direct cytotoxicity, Tarantini *et al*.^31^ tested 15 nm and 55 nm sized SiNPs in human intestinal cell lines and observed significantly increased secretion of inflammatory cytokines by the cells. In another report, this cytotoxic potential of SiNPs was adopted as potential effective strategy for anti-cancer treatment^32^.

However, the cytotoxicity of SiNPs appears to be highly dependent on size, dose, cell types, and route of administration^11^,^12^,^33^. This is why some studies reported no significant toxicity by SiNPs, others reported more toxicity by smaller sizes of SiNPs (less than 50 nm), and the others reported more toxicity by larger sizes of SiNPs (more than 100 nm)^34^,^35^,^36^. Recently, Zhao *et al*.^35^ reported that the size of silica nanoparticle is important in the interaction between nanoparticles and cell membranes. Larger sized SNPs (>100 nm) can induce significant distortion of the cell membrane and eventual cell rupture whereas smaller sized SiNPs can penetrate cell membranes without membrane rupture^35^. The dose dependent cytotoxicity of SiNPs is a well known phenomenon^11^,^33^,^37^. Because the toxicity of SiNPs depends on cell types, the toxicity found in one organ system cannot be totally applied to another organ system such as the eye that has sophisticated anatomy and various cell types. As of now, it is rare to find studies investigating SiNPs toxicity in ocular system. In one report, intravitreal injection of SiNPs induced no significant toxicity in mice^13^.

Our study confirms that SiNPs of three different sizes (50, 100, 150 nm) successfully penetrated cell membranes of HCECs and had no significant cytotoxic effect on cultured HCECs. There was no significant difference in cellular uptake and proportion of apoptosis as a function of SiNP size in this study. In addition, cytoplasm and nuclear membrane damage were independent of SiNP size as well. As mentioned earlier, HCEC is the first encounter cells of topically instilled eye drop. It is already known that SiNPs can induce oxidative stress and autophagy when cultured with various types of cells^37^,^38^,^39^. The increased ROS generation observed in our study is consistent with previous reports. SiNPs induced a mild increase of ROS and the viability of HCEC at concentrations up to 100 μg/ml. Cellular oxidative stress or ROS generation can be a useful predictor of SiNPs induced nanotoxicity^40^,^41^,^42^. However, we found the cell viability of HCECs was not affected significantly despite the mild elevation of ROS with SiNPs addition. Reactive oxygen species (ROS) is a collective term that includes oxygen radicals and non-radical derivatives of molecular oxygen such as hydrogen peroxide^43^. It is important that ROS is continuously generated from the mitochondrial electron transfer chain reaction and certain amounts of ROS are essential for cellular signal transduction and cellular homeostasis in normal physiologic states^43^. Therefore, a mild increase of ROS could protect cells against apoptosis and induces cell survival while abnormal high concentrations of intracellular ROS inevitably induces necrosis or apoptosis^44^. We think the mild increase of ROS played no significantly negative role in cell viability in HCECs.

The unaffected cell viability was further verified by the mTOR pathway and the autophagy in this study. It has been known that mTOR pathway is a key regulator of cell survival. When mTOR (phosphorylated mTOR is activated mTOR) is activated, the apoptotic pathway is inhibited and instead, cellular protein synthesis is activated for cell division and survival. In addition, activation of mTOR is known as one of the major inhibitory pathways that induce autophagy. As well known, autophagy is a natural cellular process to clean up unnecessary and dysfunctional cellular components for recycling^45^. It helps cells to overcome external stress and to promote survival in harsh environments. However, recently, several reports indicated that mTOR pathway is not the only control mechanism of autophagy. Autophagy can be regulated by various pathways which are independent of mTOR, and this suggests that these two pathways (mTOR and autophagy) are not always coupled to each other^46^,^47^,^48^,^49^. The uncoupling of mTOR and autophagy was observed in our study. Our data suggest that mTOR activation and intact autophagy can co-exist in HCEC with intracellular SiNPs accumulation. The activation of cellular survival machinery may be stimulated by cleaning up of intracellular debris including SiNPs.

In our study, SiNPs distribution was localized mainly in cytoplasmic vacuoles with no intra-nuclear invasion. This finding is consistent with previous reports^11^,^50^. The main mechanism of cellular uptake for nanoparticles is known as endocytosis^12^,^36^. Endocytosed SiNPs can induce cellular structural damage as previously reported^11^. In NIH/3T3 cells, co-culture with SiNPs induced dose dependent internalization of SiNPs, significant damage of intracellular structure (with 200 μg/ml of 20 nm sized SiNPs), and mitochondrial cristae destruction (with 10 μg/ml of 60 nm sized SiNPs). Reversible mitochondrial damage by SiNPs was also reported^51^. However, we could not find any significant destruction of intracellular structure including mitochondria. This discrepancy may be due to the different cell types tested. As previously discussed, cytotoxicity by SiNPs are cell type dependent. Another interesting finding is the existence of SiNPs inside autophagosome in HCEC. This finding may indicate the active control of intracellular SiNPs by HCECs to avoid possible cellular damage.

There are some limitations in this study. The first limitation is the lack of *in vivo* experiments. Although sufficient safety was implied *in vitro*, the result is not always repeatable *in vivo* because of many confounding factors in the human ocular surface. Therefore, a further study using *in vivo* model is necessary to confirm the safety issue of SiNPs in ocular surface. The second, we tested concentrations up to 100 μg/ml for the SiNPs. It is possible that higher concentration of SiNPs can induce cellular damage that was not detectable in lower concentration. In fact, we observed significant cellular toxicity with SiNPs with 100 mg/ml concentration (data not shown). However, we think the tested concentrations (25, 50, and 100 μg/ml) in our study are reasonable when considering future clinical applications of SiNPs for ocular topical drug delivery.

There are certainly more safety issues to be addressed regarding SiNPs for specific ophthalmic applications. The safe biodegradation of SiNPs is one of them. While many organic compounds can be degraded inside the eye, some inorganic compounds, such as gold and other metals, can persist in tissue without breaking down. Additionally, the potential effect of surface charge of SiNPs on intraocular distribution and biodegradation is unknown. The surface chemical modification can significantly alter the stability and degradation rate of silicon crystals inside the eye^52^. Furthermore, the elimination process of intraocular SiNPs should be elucidated through a further investigation. It is known that most intraocular drugs are cleared from the eye either via aqueous outflow pathway or trans-retina/choroid pathway. In a previous study, the aqueous outflow pathway, not trans-retina/choroid pathway, was the main clearance route of porous silicon micro-particles after intravitreal injection^53^. Therefore, we hypothesize that the elimination of SiNPs from ocular tissue may also follow the aqueous humor outflow pathway.

In conclusion, we confirmed the safety of SiNPs with sizes of 50, 100, and 150 nm in cultured HCECs with concentrations up to 100 μg/ml. Cellular uptake of SiNPs were localized to cytoplasm with significant activation of mTOR and autophagy. The overall cell viability was not affected significantly by SiNPs in HCECs. These findings can be the pioneering step for successful topical ophthalmic use of SiNPs for drug or gene delivery.

## Methods

We confirm that all mandatory laboratory health and safety procedures were complied with in the course of conducting all experimental work reported here.

### Nanoparticle synthesis and characterization

SiO_2_ nanoparticles (SiNPs; sizes: 50, 100, 150 nm) were prepared using the Stöber synthesis method following the previous study^54^. Tetraethylorthosilicate (TEOS, Samchun), ethyl alcohol (EtOH, anhydrous, 99.5%, Daejung, Kyeonggi, Korea), and ammonia solution (NH_4_OH, 28%, Junsei, Tokyo, Japan) were used as materials. To synthesize 50 nm of SiNPs, 2 mL of ammonia and 50 mL of EtOH were first mixed and then 1 mL of TEOS was added to the solution. 100 and 150 nm of SiNPs were prepared by equal molar ratio. 1.5 mL of TEOS was added to the as-prepared 3 mL solution of ammonia in 50 mL of ethyl alcohol. Smaller sized SiNPs could be produced by quickly adding TEOS while stirring the solution. Afterward, the solutions were stirred for 12 h at the ambient condition (25 °C, 1 atm). The prepared SiNPs were washed three times with EtOH using centrifugation (10,000 rpm, 15 min). The final SiNPs precipitates were dispersed in distilled water.

The surface charge of the prepared SiNPs was measured by the zeta potential (SZ-100, Horiba) in distilled water and Dulbecco’s Phosphate-Buffered Saline (DPBS). The size and distribution were analyzed by scanning electron microscopy (SEM) (SIGMA, Carl Zeiss) images and ImageJ software. The dispersity of the nanoparticle was defined as the coefficient of variation (Dispersity (%) = σ/*d* × 100, where σ is the standard deviation and *d* is the mean size)^14^.

### Cell culture

The human corneal epithelial cells (HCEC) (catalog number: PCS-700–010) were purchased from American Type Culture Collection (ATCC; Rockville, MD, USA). Cells were resuspended in corneal epithelial cell basal medium supplemented with a growth kit supplied by ATCC. The cells were plated in 75 cm^2^ tissue flasks, and then were maintained at 37 °C in a 5% CO_2_ and 95% air humidified atmosphere. Culture medium was changed every three days and the cells were passed using 0.05% Trypsin-EDTA (Gibco BRL, CA, USA), and cell with passage number ≤5 are used in this study.

### Treatment of Silica nanoparticles (SiNPs)

The 50 nm, 100 nm and 150 nm of SiNPs were confirmed using scanning electron microscope (SEM). The stock solutions of SiNPs were 10 mg/mL in Dulbecco’s phosphate-buffered saline (DPBS; Gibco) and all particles were sonicated for 30 min before mixing into culture media.

### Electron Microscopy and Ultrastructural Analysis

For the transmission electron microscopic (TEM) observations, HCECs which were treated three sizes of SiNPs for 24 h, then fixed in 3.7% paraformaldehyde (Sigma Aldrich) and 2.5% glutaraldehyde (Sigma Aldrich) in 0.1 M phosphate buffer (PB; pH7.6) for overnight. After washing in 0.1 M PB, HCECs were post-fixed with 1% osmium tetroxide (OsO_4_) in same buffer for 1 h. Then the cells were dehydrated with a series of the graded EtOH (Merk, Kenilworth, NJ, USA). The cells were embedded in Epon 812 and then polymerization was performed at 60 °C for 3 days. Ultrathin sections (60~70 nm) were obtained by ultramicrotome (Leica Ultracut UCT, Germany). Ultrathin sections collected on grids (200mesh) were examined under the transmission electron microscope (JEM-1010; JEOL, Tokyo, Japan) operating at 60 kV and images were recorded by the CCD camera (SC1000, Gatan, USA). The length on the electron micrograph was measured using GMS software (Gatan, USA). Normal control was incubated only corneal basal medium for 24 h and positive control for autophagy was treated 50 μM chloroquine diphosphate for 24 h.

### Measurement of reactive oxygen species (ROS)

Generation of ROS was detected using OxiSelect *In Vitro* ROS/RNS Assay Kit (catalog number: STA-347: Cell Biolabs. Inc., San Diego, CA, USA). HCEC were treated with each size of SiNPs at different concentrations (0, 25, 50, 100 μg/ml) for 24 h and 48 h. Following incubation, supernatants and cells were collected separately. Sonicated cells and the supernatants were assayed for the measurement of intracellular and extracellular ROS respectively following the manufacturer’s protocol. Hydrogen peroxide (20 μM) was used to generate a standard curve by serial dilution and 50 μL of appropriate samples from cells or supernatants were transferred to a 96-black well plate. 50 μL of Catalyst and 100 μL of dichloro-dihydro-fluorescein diacetate (DCFH) solution were added in order and the plate was incubated at room temperature for 30 min. Finally, the fluorescence was measured at 480 nm excitation/530 nm emission.

### Total Glutathione (GSSG/GSH) Assay

Glutathione was measured with the OxiSelect^™^ Total Glutathione Assay Kit (catalog number: STA-312: Cell Biolabs, INC.). HCECs were treated with each size of the SiNPs at different concentrations (0, 25, 50, 100 μg/ml) for 24 h and 48 h. Following incubation, trypsinized cell lysates were sonicated and resuspended in 0.5% metaphosphoric acid. The prepared kit reagents were added step by step according to the manufactural protocols. Finally, the microplate reader was used for a kinetic assay and the absorbance was measured at 405 nm. The plate was measured at 1 min intervals for 10 min and concentrations were calculated.

### Cell Viability Assay

Cell viability assays were performed using cell counting kit (CCK-8) reagent (Dojindo Molecular Technologies, Inc. Kumamoto, Japan) according to the manufacturer’s protocol. Briefly, HCECs were cultured at 1 × 10^4^ cells/well in a 96-well plate and incubated for 24 h. Following the adherence of cells, 50 nm, 100 nm, and 150 nm SiNPs were added to the culture media for 24 h and 48 h, dose-dependently; 0, 25, 50, 100 μg/ml. After the appropriate incubation, 10 μL of CCK-8 solution was added to each cultured well and the absorbance was measured at 450 nm after 2 h-incubation of HCECs with the reagent.

### Lactate Dehydrogenase (LDH) assay

Cellular death by membrane damage was measured using LDH cytotoxicity detection kits (Takara Bio Inc., Shiga, Japan). The experimental procedures were according to the manufacturer’s protocol. Briefly, HCECs were cultured at 1 × 10^4^ cells/well in a 96-well plate and incubated for 24 h. Following the adherence of cells, 50 nm, 100 nm, and 150 nm SiNPs were treated to cells for 24 h and 48 h, dose-dependently; 0, 25, 50, 100 μg/ml. For positive controls, the maximum release of LDH was triggered by 1% triton X-100 solutions. Wells with culture media only and no cells were used as negative controls. Following the incubation of cells, all supernatants were transferred into a new 96-well plate and the reaction mixture was added followed by incubation for 20 min at room temperature. Absorbance was measured at 490 nm.

### Terminal Deoxynucleotidyl Transferase (TUNEL) assay

For detection of fragmented DNA due to apoptosis at the cellular level in HCEC, TUNEL assay was performed using the APO-BrdU^TM^ TUNEL assay kit (catalog number: A23210: Molecular Probes, Eugene, OR, USA) according to the manufacturer’s protocol. All SiNPs- treated HCECs were fixed using 1%-paraformaldehyde and were washed with PBS. Cells in ice-cold 70%-ethanol were incubated in a −20 °C freezer for 18 h, then were labeled using TdT enzyme and anti-BrdU mixture solution. Finally, propidium iodide and RNase A staining buffer were added to the cells and samples were analyzed by flow cytometry, Calibur (BD Biosciences, San Jose, CA, USA).

### Western Blot Analysis

All SiNPs treated HCECs were lysed in ice-cold RIPA buffer (50 mM Tris-HCl (pH 8.0), 150 mM NaCl, 1% NP-40, 0.5% deoxycholate, and 0.1% SDS) for 30 min. The debris was removed by centrifugation at 16,000 *g* for 1 min. Equal amounts (20 μg) of total cell protein were separated by SDS-polyacrylamide gel electrophoresis (SDS-PAGE), and transferred to a PVDF membrane. After blocking with 5% BSA in TTBS buffer (10 mM Tris, pH 8.0, 150 mM NaCl, 0.1% Tween20) for 1 h at room temperature, membranes were incubated overnight at 4 °C with the following primary antibodies: rabbit anti-LC3A/B (1:1,000; catalog number: 12741; Cell Signaling, Beverly, MA, USA), rabbit anti-phospho-mTOR (1:1,000; catalog number: 5536; Cell Signaling), rabbit anti-mTOR (1:1,000; catalog number: 2983; Cell Signaling) and mouse anti-β-actin (1:10,000; catalog number: sc-47778; Santa Cruz, Biotechnology, Dallas, Texas, USA). The membranes were incubated with peroxidase-conjugated secondary antibody for 1 h at room temperature. Blots were developed using an enhanced chemiluminescence (ECL) kit (catalog number: RPN2232; GE healthcare, Buckinghamshire, UK) and visualized using Fujifilm Image Reader LAS-3000 (Fujifilm, Tokyo, Japan). Each experiment was repeated at least 3 times, and densitometric analysis was performed using the Multi Gauge V3.0 (Fujifilm Life Science, Tokyo, Japan).

### Immunocytochemistry

HCECs were seeded at a density of 3 × 10^4^ cells per milliliter and grown on 4-well Lab-Tek chamber slides (Nalgene Nunc Penfield, NY, USA) and 0, 25, 50, 100 μg/mL of SiNPs were treated for 24 h. Cells were fixed with 3.7% paraformaldehyde for 10 min at room temperature (RT) and permeabilization was carried out using 0.1% triton x-100 for 5 min at RT. Following washing steps with DPBS, cells were blocked using 1% bovine serum albumin (BSA) in DPBS for 30 min at RT. The chamber slides were incubated overnight at 4 °C with rabbit polyclonal anti-LC3B (0.5 μg/mL; catalog number: L10382; Molecular Probes). The chamber slides were then washed with DPBS and incubated with Alexa488-conjugated donkey anti-rabbit antibody (1:1000; catalog number: A21206; Molecular Probes) for 2 h at room temperature. Staining for F-actin was carried out using tetramethylrhodamine isothiocyanate (TRITC)-conjugated phalloidin (1 μg/mL; Sigma-Aldrich). Counterstaining of cell nuclei was carried out using 4′,6-diamidino-2′-phenylindole (DAPI, catalog number: P36931; Molecular Probes) with mounting solution. Slides were viewed under the fluorescence microscope.

### Statistical Analysis

Data are presented as mean ± standard error and the statistical significance was determined by one-way analysis of variance (ANOVA) followed by the Dunnett’s multiple comparison test. P values less than 0.05 were regarded as significant using GraphPad Prism Ver. 5.01 (GraphPad Software Inc., La Jolla, CA, USA).

## Additional Information

**How to cite this article**: Park, J.-H. *et al*. The Effect of Silica Nanoparticles on Human Corneal Epithelial Cells. *Sci. Rep.* **6**, 37762; doi: 10.1038/srep37762 (2016).

**Publisher's note:** Springer Nature remains neutral with regard to jurisdictional claims in published maps and institutional affiliations.

## Figures and Tables

**Table 1:** The size and zeta potential of silica nanoparticles investigated in this study.

Size	Distilled water			DPBS
	Diameter (nm)	Zeta potential (mV)	Dispersity (%)	Zeta potential (mV)
50 nm	50.68 ± 2.93	−56.63 ± 3.70	5.79	−3.77 ± 1.36
100 nm	102.81 ± 3.78	−74.67 ± 1.00	3.68	−2.30 ± 1.47
150 nm	149.41 ± 8.39	−75.87 ± 3.20	5.62	−6.90 ± 1.51

Data presented as mean ± standard deviation.

Abbreviation: DPBS (Dulbecco’s Phosphate-Buffered Saline).
